# Wildfire‐induced short‐term changes in a small mammal community increase prevalence of a zoonotic pathogen?

**DOI:** 10.1002/ece3.5688

**Published:** 2019-10-25

**Authors:** Frauke Ecke, Seyed Alireza Nematollahi Mahani, Magnus Evander, Birger Hörnfeldt, Hussein Khalil

**Affiliations:** ^1^ Department of Wildlife, Fish, and Environmental Studies Swedish University of Agricultural Sciences Umeå Sweden; ^2^ Department of Clinical Microbiology, Virology Umeå University Umeå Sweden; ^3^ Institute of Integrative Biology University of Liverpool Liverpool UK

**Keywords:** amplification effect, bank vole, demography, disturbance, prevalence, puumala hantavirus

## Abstract

Natural disturbances like droughts and fires are important determinants of wildlife community structure and are suggested to have important implications for prevalence of wildlife‐borne pathogens. After a major wildfire affecting >1,600 ha of boreal forest in Sweden in 2006, we took the rare opportunity to study the short‐term response (2007–2010 and 2015) of small mammal community structure, population dynamics, and prevalence of the Puumala orthohantavirus (PUUV) hosted by bank voles (*Myodes glareolus*). We performed snap‐trapping in permanent trapping plots in clear‐cuts (*n* = 3), unburnt reference forests (*n* = 7), and the fire area (*n* = 7) and surveyed vegetation and habitat structure. Small mammal species richness was low in all habitats (at maximum three species per trapping session), and the bank vole was the only small mammal species encountered in the fire area after the first postfire year. In autumns of years of peak rodent densities, the trapping index of bank voles was lowest in the fire area, and in two of three peak‐density years, it was highest in clear‐cuts. Age structure of bank voles varied among forest types with dominance of overwintered breeders in the fire area in the first postfire spring. PUUV infection probability in bank voles was positively related to vole age. Infection probability was highest in the fire area due to low habitat complexity in burnt forests, which possibly increased encounter rate among bank voles. Our results suggest that forest fires induce cascading effects, including fast recovery/recolonization of fire areas by generalists like bank voles, impoverished species richness of small mammals, and altered prevalence of a rodent‐borne zoonotic pathogen. Our pilot study suggests high human infection risk upon encountering a bank vole in the fire area, however, with even higher overall risk in unburnt forests due to their higher vole numbers.

**Open Research Badges:**

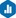

This article has earned an Open Data Badge for making publicly available the digitally‐shareable data necessary to reproduce the reported results. The data is available at https://osf.io/6fsy3/.

## Graphical abstract

Natural disturbances, such as droughts and fires are important determinants of wildlife community structure, are increasing due to climate change and are suggested to have important implications for the prevalence of wildlife‐borne pathogens. In a comprehensive postfire field study using an ecosystem approach, we show that the generalist bank vole recovered/recolonized forest areas quickly with cascading effects on the community structure of small mammals as well as on the prevalence of a rodent‐borne zoonotic pathogen and human infection risk.

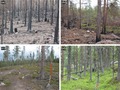

## 1 INTRODUCTION

Natural disturbances like droughts and wildfires are often weather‐induced and have shaped ecosystems and wildlife communities globally. At northern latitudes, the frequency and extent of extreme weather events are predicted to increase with climate change (IPCC, 2014). In summer, this will result in long periods of high temperatures in combination with low precipitation (Francis & Skific, 2015), increasing the frequency and intensity of droughts and forest fires.

Many species living in areas recurrently struck by natural disturbances are evolutionary well adapted to such events (Esséen, Ehnström, Ericson, & Sjöberg, 1997; Humphries & Baldwin, 2003). In the long‐term, disturbances like forest fires promote biodiversity (Kelly & Brotons, 2017) and are even a prerequisite for the development of communities typical for e.g., the boreal region (Esséen, Ehnström, Ericson, & Sjöberg, 1992; Zackrisson, 1977). Forest fires frequently favor species that are specialized to exploit resources exclusively provided by the disturbance (e.g., large amounts of dead wood; Buddle, Langor, Pohl, & Spence, 2006). However, in the short‐term and among small mammals, disturbances in general and forest fires in particular favor generalist species (Griffiths & Brook, 2014; Shenko, Bien, Spotila, & Avery, 2012) that often host multiple pathogens (as evident from the hyperservoirs identified by Han, Schmidt, Bowden, & Drake, 2015). Among rodents, for example, the generalist deer mouse (*Peromyscus maniculatus* Wagner) is the predominant species in North American forests frequently experiencing fires (Krefting & Ahlgren, 1974; Roberts, Kelt, Wagtendonk, Miles, & Meyer, 2015). The habitat generalist Merriam's kangaroo rat (*Dipodomys merriami* Mearns; Timm, Álvarez‐Castañeda, & Lacher, 2016) that occurs in the southwestern United States and northern Mexico is largely favored by fires in semidesert grass‐shrublands (Monasmith, Demarais, Root, & Britton, 2010).

Disturbances also have major impacts on pathogen prevalence in wildlife, but it remains inconclusive if disturbance increases or decreases prevalence, transmission among animals, and/or potential transmission to and infection risk of humans. In the short‐term, forest fires significantly reduce richness of helminths in the long‐tailed field mouse *Apodemus sylvaticus* Linnaeus, increase prevalence of monoxenous (life‐cycle restricted to a single host species) helminths but decrease prevalence of heteroxenous helminths (life‐cycle dependent on multiple host species; Torre, Arrizabalaga, Feliu, & Ribas, 2013). The latter response is likely caused by intermediate hosts being rare or absent in intensively burnt forests (Torre et al., 2013). Habitat disturbance by off‐road vehicles and habitat fragmentation are associated with high prevalence of Sin Nombre virus (SNV) in deer mice and an increased encounter rate among hosts is the suggested mechanism (Langlois, Fahrig, Merriam, & Artsob, 2001; Mackelprang, Dearing, & Jeor, 2001). However, if disturbed areas function as dispersal sinks for juveniles (that might be uninfected in case of certain horizontally‐spread infections; Kallio et al., 2010), pathogen prevalence might decrease (Calisher et al., 2001; Lehmer, Clay, Pearce‐Duvet, St. Jeor, & Dearing, 2007). The concept of the dilution effect predicts that a high proportion of noncompetent hosts (dead ends) occurring in diverse animal communities reduces disease risk (Ostfeld & Keesing, 2000; Schmidt & Ostfeld, 2001). Hence, if a disturbance favors competent host species at the expense of noncompetent hosts, we can expect an increase in pathogen prevalence, while we expect the opposite effect if a disturbance favors noncompetent hosts. Ultimately, the direction of the response of small mammals and their pathogens to forest fires is likely driven by the severity and spatial extent of the disturbance and shows likely species‐specific responses.

Postfire habitat patchiness (especially presence of unburnt forest patches) and postfire availability of food resources are important drivers of the response of small mammals to forest fires. Unburnt forest patches can act as source habitats and are likely to contribute to fast recolonization of long‐tailed field mice (Monimeau, Mouillot, Fons, Prodon, & Marchand, 2002). Fire severity has a significant impact on plant survival and recovery (Schimmel & Granström, 1996), which in turn has cascading effects on recolonization of burnt patches by small mammals. Forest fires in coniferous forests might favor granivorous species since e.g., many pine species (*Pinus* spp.) shed their seeds as a response to forest fires (Daskalakou & Thanos, 1996; Habrouk, Retana, & Espelta, 1999). Also graminivorous small mammals might recolonize burnt forests within the first postfire years and might even be fire‐favored. Different grass species (e.g., *Deschampsia flexuosa* (L.) Trin.) are known for their fast recovering capacity after forest fires as long as burn depth does not destroy rhizomes (Schimmel & Granström, 1996). Insects such as weevils are strongly favored by forest fires (Johansson, Andersson, Hjältén, Dynesius, & Ecke, 2011), and since they are frequently found on the ground, forest fires are expected to also favor insectivores and/or small mammals with a broad food niche.

In our pilot study, we took the rare opportunity of a major wildfire (>1,600 ha) in a boreal forest area in northern Sweden in 2006 to study the short‐term response (2007–2010 and 2015) of small mammal community structure, population dynamics of small mammals, and pathogen prevalence. The target pathogen in our study was the Puumala orthohantavirus (PUUV), a single‐stranded RNA virus with bank vole (*Myodes glareolus* Schreber) as the only reservoir host (Brummer‐Korvenkontio et al., 1980). PUUV is horizontally transmitted through physical contact between voles. Inhalation of viral particles from these excretions is the dominant pathway of human exposure, causing nephropatia epidemica, a hemorrhagic fever with renal syndrome in humans (Olsson et al., 2003; Vapalahti et al., 2003). PUUV can remain infectious in the environment for several weeks (Kallio et al., 2006). We hypothesized that the fire‐favored insectivorous shrews (*Sorex* spp.) graminivorous field voles (*Microtus agrestis* Linnaeus) and bank voles. If shrews and field voles are fire‐favored, we should expect lower PUUV prevalence in the fire area compared to unburnt forests due to a dilution effect, while PUUV prevalence in the fire area should be high if the disturbance mainly favored bank voles. We expect the forest fire to increase patchiness of food resources and/or habitat features that provide protection from predators. Therefore, susceptible hosts will aggregate in these patches, which should increase transmission risk and ultimately PUUV prevalence.

## 2 MATERIAL AND METHODS

### 2.1 Study area

**Figure 1:**
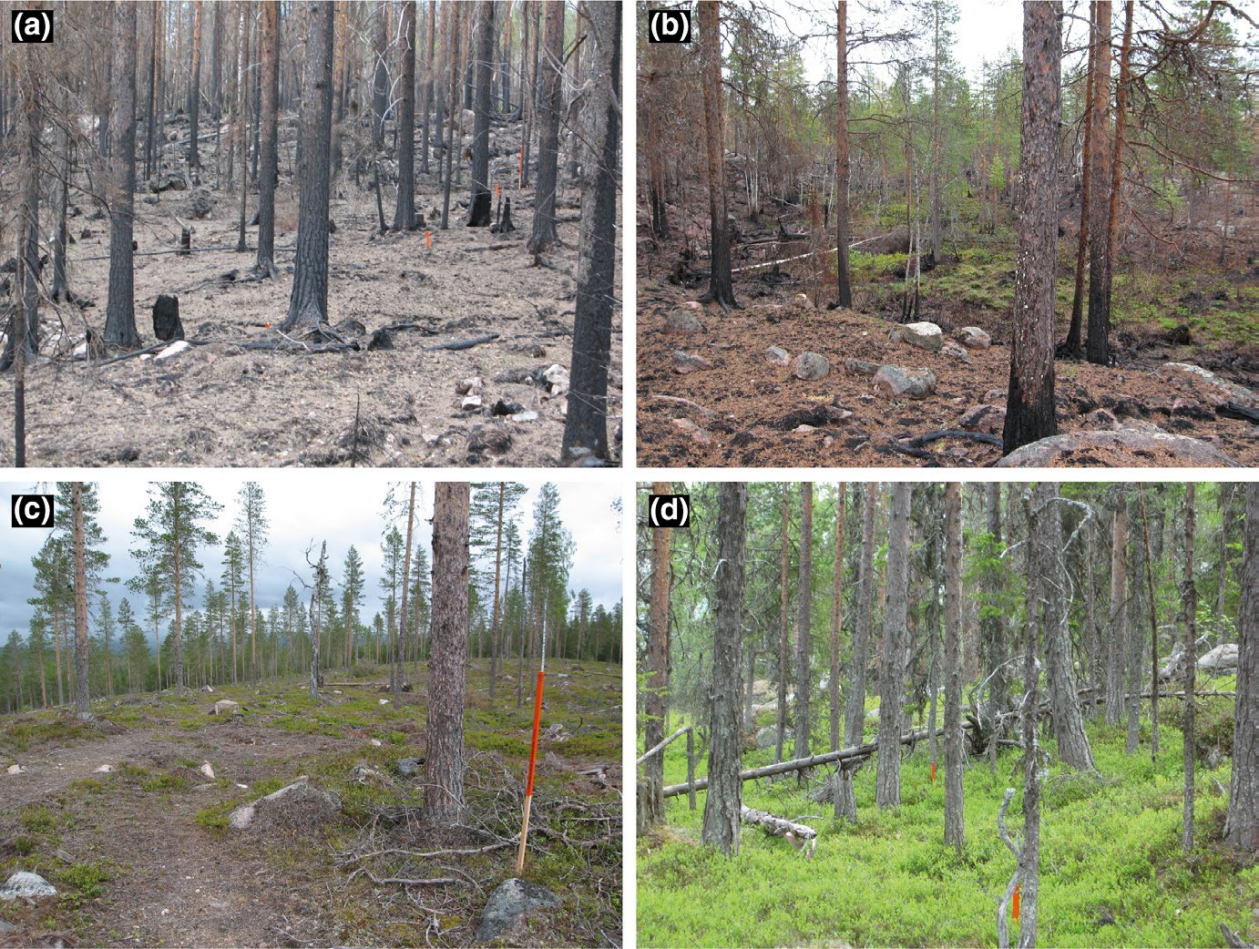
The studied forest types (a, b) fire area, (c) clear‐cut, and (d) unburnt forest in early June 2007. The forest fire in August 2006 was severe and much of the field layer and soil horizon was burnt (a), but in some moist patches vegetation either survived or recovered, at least partly, already during the first postfire year (b). In (a), (c, d) the sticks mark trapping stations for small mammals. Photography credit: Frauke Ecke

We performed our pilot study in a boreal forest landscape in northern Sweden near the village of Bodträskfors (approximately 66°9′N 20°49′E). Boreal Sweden is dominated by coniferous forests that have a long fire history, but with fires being generally rare since the early 20th century due to fire suppression (Niklasson & Granström, 2000). In 2006 (11 August–8 September), a 1,628‐ha forest area burnt severely, destroying much of the top soil and resulting in 100% tree mortality in large parts of the area due to fire consumption of both trees and tree roots (Figure 1). Coniferous forest of dwarf‐shrub and lichen types dominated the prefire forest area with Scots pine (*Pinus sylvestris* Linnaeus) being the most common tree species (60% of area), with incidence of Norway spruce (*Picea abies* (L.) H. Karst.) (25%) and deciduous species (15%) (Johansson et al., 2011).

The small mammal community (considering voles, lemmings, mice, and shrews) in lowland forests of northern Sweden is rather species poor, with a species pool of at most 10 species, including the bank vole, gray‐sided vole (*Myodes rufocanus* Sundevall), field vole, wood lemming (*Myopus schisticolor* Liljeborg), European water vole (*Arvicola amphibius* Linnaeus), and five shrew species including common shrew (*Sorex araneus* Linnaeus). However, of these, only the bank vole, field vole and common shrew are common in lowland forests in the region (Ecke, Löfgren, & Sörlin, 2002). Rarely more than 3–4 species (mostly 1–2 species) are found in 1‐ha trapping plots as indicated by the National Environmental Monitoring Program of Small Rodents (Ecke & Hörnfeldt, 2018). The density of the gray‐sided vole in lowland forests has significantly declined during the last decades (Ecke et al., 2010; Hörnfeldt, 2004; Magnusson, Hörnfeldt, & Ecke, 2015), the water vole is more common close to human settlements, river banks and grasslands (Batsaikhan et al., 2016), and the wood lemming is a habitat specialist restricted to coniferous forests rich in mosses (Ims, Bondrup‐Nielsen, Fredriksson, & Fredga, 1993).

### 2.2 Small mammal survey

**Figure 2:**
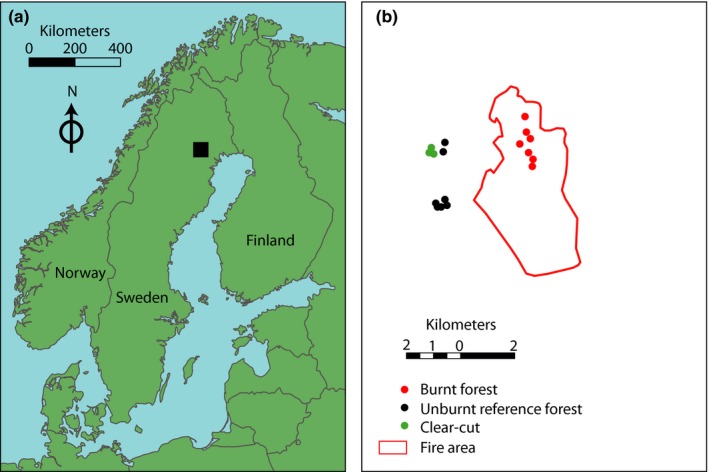
Location of the study area in northern Sweden (a) as well as of the fire area and the trapping plots in the fire area, unburnt reference forests, and clear‐cuts (b)

We surveyed small mammals in a total of 17 1‐ha trapping plots (altitude 99–242 m above msl; Figure 2), viz. in the fire area (*n* = 7; >100‐year‐old forest before the fire), in unburnt reference forests (*n* = 7; >100‐year‐old) of the same vegetation type and similar tree composition as in the fire area, and clear‐cuts (<3 years old; *n* = 3). We included clear‐cuts since clear‐cutting is the most pronounced artificial disturbance in boreal forests (Ecke, Magnusson, & Hörnfeldt, 2013; Zwolak, 2009), while forest fires have been the dominating natural disturbance until the start of fire suppression (Esséen et al., 1992; Niklasson & Granström, 2000). In addition, clear‐cutting might result in similar habitat structure as induced by forest fire, e.g., in terms of woody debris (if at least partly left on‐side) and altered vegetation in field layer. The mean Euclidean distance between trapping plots in the fire area and in unburnt reference forests was 3.1 km (minimum 2.8 km) and that between trapping plots in the fire area and in clear‐cuts was 3.5 km (minimum 3.2 km), while the distance between trapping plots in unburnt forests and in clear‐cuts was shorter (Figure 2). The movement distance of bank voles rarely exceeds 900 m (Andrzejewski, Babinska‐Werka, Liro, Owadowska, & Szacki, 2000; Kozakiewicz, Chołuj, & Kozakiewicz, 2007), even though long‐distance movement of up to 3 km has been observed in voles (Oksanen, Schneider, Rammul, Hambäck, & Aunapuu, 1999). Our main focus was on the comparison between the fire area and unburnt reference forests on one hand and on the comparison between the fire area and clear‐cuts. Considering potential movement distance and our study design, we consider trapping plots in the fire area to be independent of those in the unburnt reference forests and clear‐cuts.

Each 1‐ha plot consisted of 10 trap stations with five traps each, centered and spaced 10 m apart along the diagonal of the 1‐ha plot (Hörnfeldt, 1978, 1994). In each plot, we snap‐trapped small mammals twice per year, in spring (mid‐June) and autumn (mid‐end September) for three consecutive nights. We performed the survey in spring 2007—autumn 2010 and in spring and autumn 2015. The original task of the study was to include one complete vole cycle (hence 2007–2010) and to address potential successional effects, we also included a follow‐up in 2015. The trapping effort per ha plot and season was 150 trap‐nights with a total trapping effort of 25,500 trap‐nights for the whole study. All trapped small mammals were frozen (−20°C) within 2 hr after each trap‐night. We determined the age of the bank voles by the root length of the first mandibular molars (M_1_) following Viro (1974). As an index of density, we calculated the trapping index (number of trapped specimens per 100 trap‐nights) per species, trapping period and trapping plot.

### 2.3 Estimation of vegetation and structural habitat factors

To estimate habitat quality from the perspective of small mammals (food availability and protective cover), we estimated vegetation and structural habitat factors at each trap station within a 5 × 5 m area centered on the trap station in July 2010. Vegetation and structural habitat factors included cover of trees (>5 m height; two layers that differed in at least 5 m height) and shrubs, number of snags (standing dead trees; ≥10 cm diameter at 1 m height), number of stoneholes in the ground that can be used by the small mammals to hide (two classes: ≤5 cm diameter, >5 cm diameter), cover of epiphytic lichens on the branches of coniferous trees, ground cover of fine (≤10 cm diameter) woody debris and total length of coarse (>10 cm diameter) woody debris, cover of boulders (stones >10 cm diameter), cover of umbrella vegetation (field vegetation that has a height of ≥50 cm), cover of vegetation in the field layer (<50 cm height), mosses, lichens, bilberry (*Vaccinium myrtillus* Linnaeus), lingonberry (*Vaccinium vitis‐idaea* Linnaeus), and grasses, as well as maximum height of dwarf‐shrubs. Cover was estimated at a 5‐graded revised Braun‐Blanquet scale (1: 0, 2: >0–12, 3: >12–25, 4: >25–50, 5: >50% cover; Mueller‐Dombois & Ellenberg, 1974).

### 2.4 PUUV infection data

We analyzed lung biopsies from all bank voles by enzyme‐linked immunosorbent assay (ELISA) to detect anti‐PUUV IgG antibodies and identify sero‐positive individuals (Lindkvist, Näslund, Ahlm, & Bucht, 2008; Niklasson, Hörnfeldt, Lundkvist, Björsten, & Leduc, 1995). Shedding of PUUV is life‐long, and sero‐positivity indicates an ongoing infection in bank voles (Voutilainen et al., 2015). We therefore used the term infected instead of sero‐positive throughout this paper and calculated PUUV prevalence, i.e., the proportion of infected bank voles.

### 2.5 Statistical analyses

We performed all statistical analyses in R (R Development Core Team, 2018). To reduce the dimensionality of our 18 vegetation and structural habitat variables to a few essential components, we used a principal component analysis (PCA; prcomp function in the ggfortify package; Tang et al., 2018) on mean values of the variables per trapping plot. We used the results of the PCA (PC scores) to describe and visually distinguish the three forest types. Furthermore, we used the calculated principal component (PC) loadings of the first PC (PC1) as an explanatory variable to explain vole density and PUUV infection probability (see below).

To test the association between forest type and bank vole density, we ran generalized linear mixed models with forest type and season as well as their interactions as fixed effects and trapping plot and year as random effects with the glmer function in the lme4 package (Bates, Maechler, Bolker, & Walker, 2015). We used a Poisson error distribution function for bank vole density multiplied by 100 (to avoid decimal points). We used a *χ* ^2^‐test on observed and expected frequencies to test for differences in age structure of bank voles (14 age categories) among forest types in spring and autumn of the first postfire year and in spring and autumn aggregated for all years.

We analyzed the importance of environmental conditions on PUUV infection probability in two steps. In the first step, we evaluated if infection probability varied among forest types by applying a glmer function with a binomial (logit) error distribution with bank vole weight (model did not converge when using vole age), bank vole density, forest type, and season as well as their interaction as fixed effects and year and trapping plot as random effects. To get a mechanistic understanding of the role of forest type for infection probability, we tested in a second step and using the same model type as in the first step, the dependence of PUUV infection probability of bank voles on structural habitat variables, by also including bank vole age, bank vole density, PC1 loadings, and season as well as their interaction as fixed effects and year and trapping plot as random effects. For all models, we used an automated model selection process based on Akaike Information Criterion (AIC) using the dredge function in the MuMIn package (Barton, 2018). If the AIC score of the most parsimonious model was within 2 AIC points from the best model, we selected the former.

## 3 RESULTS

**Figure 3:**
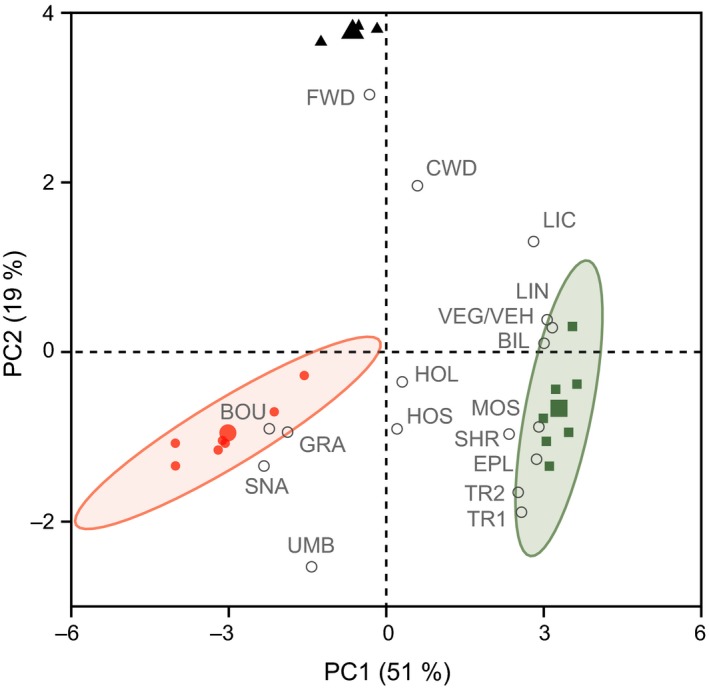
Biplot of results from principal component analysis (PCA) on structural habitat factors (white circles) in the three studied forest types (unburnt forest, green squares; fire area, red filled circles; clear‐cuts, black triangles; large symbols indicate mean values). The ellipses show the 95% confidence intervals for the respective forest types (for clear‐cuts, no confidence intervals could be calculated due to too few data points). The percentage of explained variance is given in parentheses for the two PCs. BIL cover of bilberry, BOU cover of boulders, CWD total length of coarse woody debris, EPL cover of epiphytic lichens on trees, FWD cover of fine woody debris, GRA grass cover, HOL number of large stoneholes, HOS number of small stoneholes, LIC lichen cover, LIN cover of lingonberry, MOS moss cover, TR1 cover of tree layer 1, TR2 cover of tree layer 2, SHR cover of shrubs, SNA number of snags, UMB cover of umbrella vegetation, VEG vegetation cover in the field layer, and VEH height of vegetation in the field layer. VEG and VEH yielded the same loadings

Vegetation and structural habitat factors differed significantly among the three studied forest types with unburnt forests having high cover and height of vegetation in the field layer and being rich in mosses, bilberry, and lingonberry (Figure 3; all variables except the two stonehole variables had PC scores |>0.5| along at least one PC). These forests were also characterized by multiple‐layered canopies of shrubs and trees (Figure 3). In summary, the unburnt forests were characterized by high habitat complexity. In contrast, clear‐cuts and the fire area were characterized by mainly structural habitat factors including woody debris (clear‐cuts) as well as boulders, which may be equally present but covered in vegetation in unburnt reference forests, and snags (Figure 3). Grasses recovered and benefitted in the fire area after the fire since their cover was higher in the fire area compared to both clear‐cuts and the unburnt forests (Figure 3).

**Figure 4:**
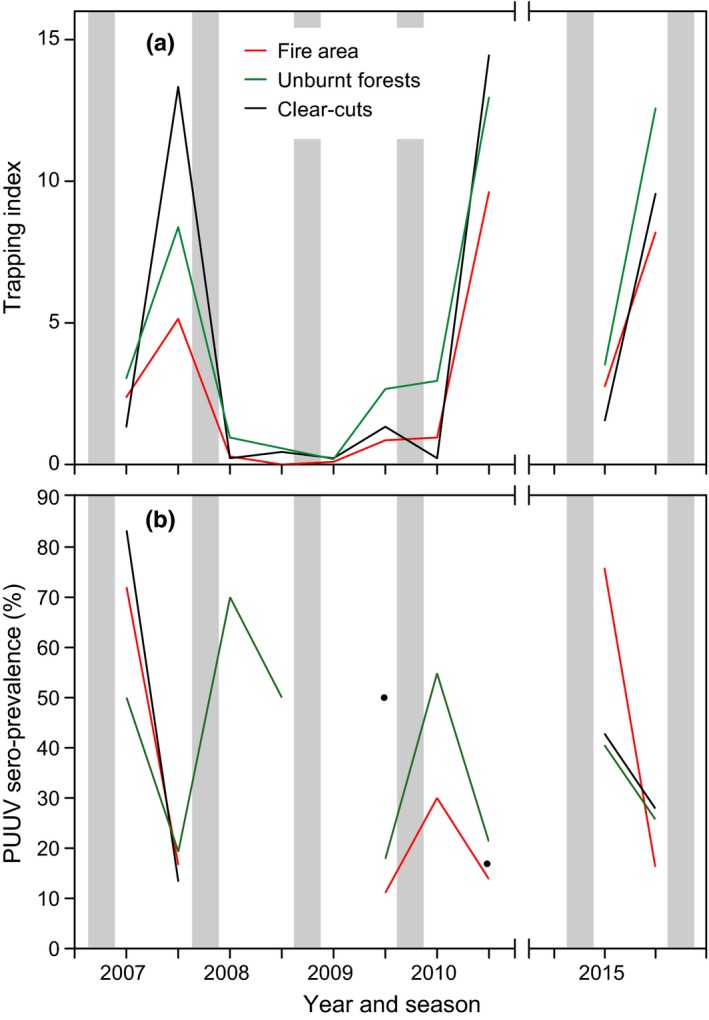
Mean trapping index of bank voles (*Myodes glareolus*) (a) and PUUV sero‐prevalence in bank voles (b) in the fire area (red line), unburnt reference forests (green line) and clear‐cuts (black line) in spring and autumn 2007–2010 and 2015. Shading indicates winter. The black filled circles in (b) show PUUV sero‐prevalence in the clear‐cut area in autumn 2009 and 2010. In (b) PUUV prevalence is shown for seasons that yielded at least five bank voles

In total, we trapped 1,012 bank voles (502 in unburnt forests, 318 in the fire area and 192 in clear‐cuts), eight gray‐sided voles (*M. rufocanus* Sundevall; five in unburnt forests, two in the fire area, and one in clear‐cuts), two wood lemmings (*M. schisticolor* Liljeborg) in unburnt forests, three yellow‐necked mice (*Apodemus flavicollis* Melchior; two in the fire area and one in clear‐cuts), and one common shrew (*S. araneus* Linnaeus) in unburnt forest. The density of bank voles showed seasonal dynamics with a 3‐year cycle with population peaks in autumn 2007, 2010, and most likely also 2015 (though only data from 1 year of this latter cycle were sampled; Figure 4a). In spring, bank vole density was highest in unburnt forests, followed by the fire area, and was lowest in clear‐cuts (Figure 4a). The autumn density of bank voles in the peak years 2007 and 2010 was highest in clear‐cuts and lowest in the burnt forests (Figure 4a). In the low phase of the complete population cycle, density was low in all three habitats, but still highest in the unburnt forests. In 2015, density was still highest in the unburnt forests (Figure 4a). These overall patterns of seasonal and habitat differences were confirmed by the generalized linear mixed model; autumn was associated with high densities in unburnt forests and clear‐cuts, whereas overall densities were low in clear‐cuts but high in unburnt forests (Table 1).

**Table 1:** Best generalized linear mixed model on the effect of forest type and season as well as their interaction (×) on bank vole density^a^

Estimate	Estimate	Standard error	*z*‐value	*p*‐value
Fixed effects				
Intercept	5.02	0.38	13.17	<.001
Clear‐cut	−0.52	0.21	−2.44	<.05
Unburnt forest	0.34	0.16	2.10	<.05
Autumn	1.15	0.02	66.47	<.001
Clear‐cut × autumn	1.02	0.04	26.65	<.001
Unburnt forest × autumn	0.06	0.02	2.75	<.01
	**Variance**	**Standard deviation**		
Random effects				
Trapping plot	0.09	0.30		
Year	0.53	0.73		

a The first principal component (PC1) of the PC analysis was included in the analysis as an explanatory variable, but was nonsignificant in the final model.

Puumala orthohantavirus prevalence of infection showed the opposite seasonal pattern compared to bank vole density, i.e., highest overall values in spring (Figure 4b). In the low phase in 2008, overall, only few bank voles were trapped (22 in total) and it was only meaningful to calculate PUUV prevalence for the specimens trapped in the unburnt forests (10 and six specimens in spring and autumn 2008, respectively). Due to low sample size, we excluded bank voles trapped in 2008 and spring 2009 from the statistical analyses. In spring of 2007, PUUV prevalence in bank voles was second highest in the fire area and in spring of 2015, it was highest in the fire area among the three forest types (Figure 4b). In autumn, however, overall, prevalence in the fire area was lowest, as was bank vole density (Figure 4a,b).

**Figure 5:**
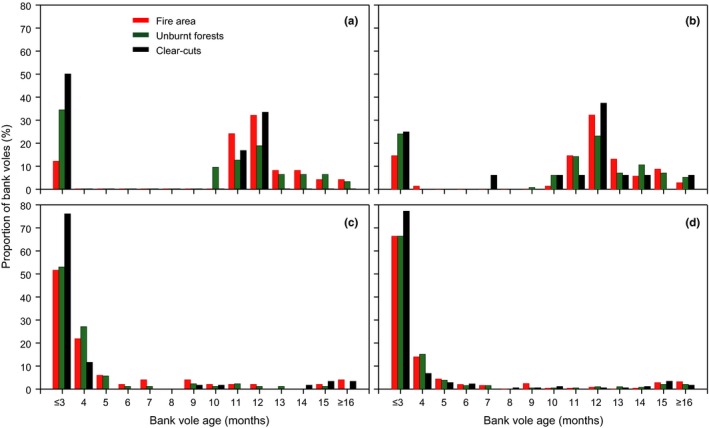
Number of bank voles (%) per age class in the three forest types for (a) first postfire spring in 2007 (fire area: *n* = 23, unburnt forests: *n* = 31, clear‐cuts: *n* = 6), (b) all springs combined (fire area: *n* = 65, unburnt forests: *n* = 111, clear‐cuts: *n* = 16), (c) first postfire autumn in 2007 (fire area: *n* = 54, unburnt forests: *n* = 86, clear‐cuts: *n* = 60), (d) all autumns combined (fire area: *n* = 247, unburnt forests: *n* = 378, clear‐cuts: *n* = 176)

The age structure of bank voles differed among the forest types and changed over time (Figure 5). In spring of the first postfire year, the fire area was dominated by overwintered breeders (≥11 months; Figure 5a; $\chi_{\text{Ref - Burn}}^{2}$ = 54.9, *df* = 13, *p* < .001; $\chi_{\text{Ref - Clear}}^{2}$ = 12.3, *df* = 13, *p* > .05; $\chi_{\text{Burn - Clear}}^{2}$ = 32.2, *df* = 13, *p* < .01), a pattern that was still evident when aggregating all spring data (Figure 5b; $\chi_{\text{Ref - Burn}}^{2}$ = 34.7, *df* = 13, *p* < .001; $\chi_{\text{Ref - Clear}}^{2}$ = 25.5, *df* = 13, *p* < .01; $\chi_{\text{Burn - Clear}}^{2}$ = 35.9, *df* = 13, *p* < .001). In contrast to the fire area, both unburnt forests and clear‐cuts had a high proportion of juveniles (≤3 months old) in spring (Figure 5a,b). In autumn of the first postfire year, clear‐cuts had a higher proportion of juveniles than the burnt and unburnt forests (Figure 5c; $\chi_{\text{Ref - Burn}}^{2}$ = 9.6, *df* = 13, *p* > .05; $\chi_{\text{Ref - Clear}}^{2}$ = 34.8, *df* = 13, *p* < .001; $\chi_{\text{Burn - Clear}}^{2}$ = 22.8, *df* = 13, *p* < .05), while there was no difference in age structure when aggregating all autumn data (Figure 5d; $\chi_{\text{Ref - Burn}}^{2}$ = 2.8, *df* = 13, *p* > .05; $\chi_{\text{Ref - Clear}}^{2}$ = 14.6, *df* = 13, *p* > .05; $\chi_{\text{Burn - Clear}}^{2}$ = 19.5, *df* = 13, *p* > .05). The weight of bank voles ≥11 months old did not differ between the fire area and unburnt reference forests (*F*_1, 178_ = 0.55, *p* > .05).

**Figure 6:**
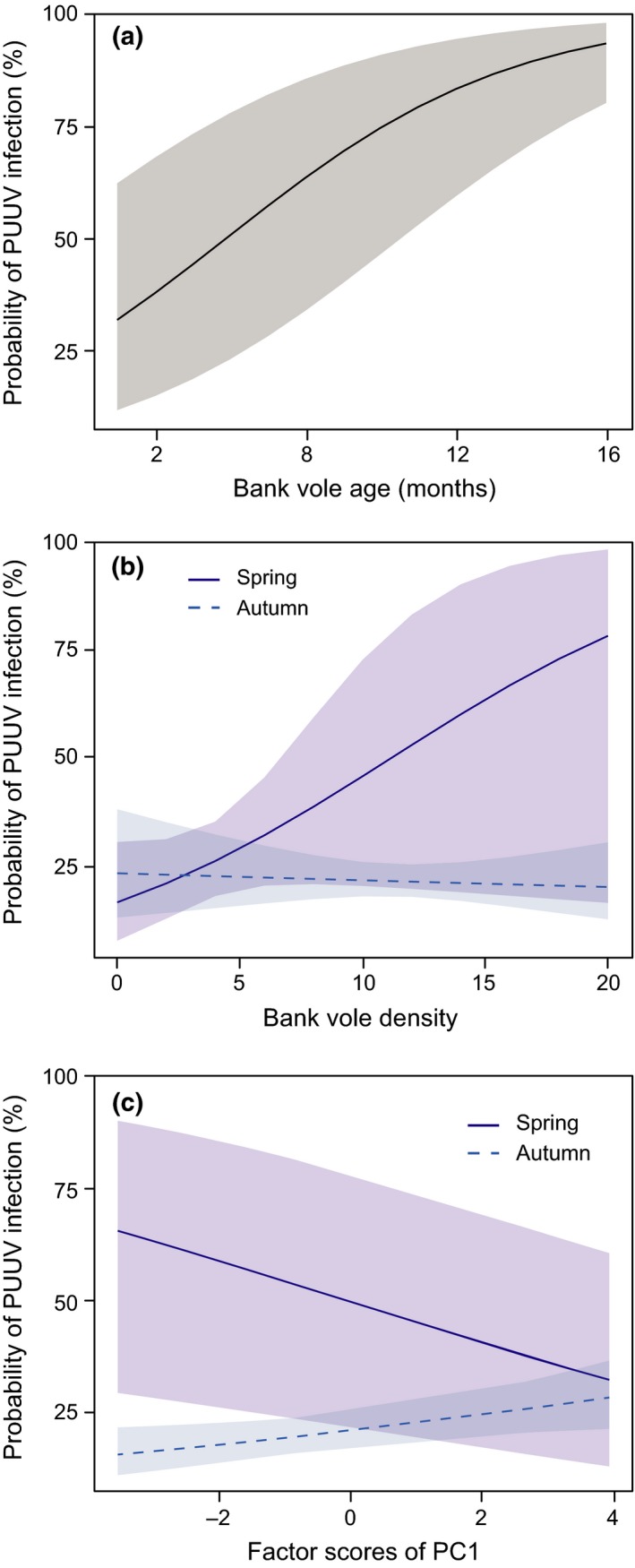
Predicted probability of PUUV sero‐positivity (probability of PUUV infection) in bank voles as a function of (a) vole age, (b) bank vole density (number of trapped bank voles per 100 trap‐nights), and (c) factor loadings of the first principal component (PC1) on the vegetation and structural habitat factors studied at each trapping plot (cf. Figure 3). High factor loadings represent heterogeneous trapping plots, rich in vegetation with multiple shrub and tree layers, while low loadings imply low vegetation cover (except grasses), high cover of boulders and high number of snags (cf. Figure 3). In (b) and (c) probability was calculated separately for spring and autumn. The shaded areas represent the 95% confidence interval of coefficient estimates

The probability of a bank vole being infected with PUUV was explained by multiple factors. Probability of infection increased with age/weight (Figure 6a, Tables 2 and 3), and was highest, despite the large variation, in spring (Figure 6b). Bank vole density was an important predictor when forest type was included in the model (Table 2) but did not appear in the final multivariate model that included habitat complexity instead (Table 3). Infection probability was overall lower in unburnt reference forests (Table 2) and in spring it was higher at lower levels of habitat complexity (as revealed by vegetation and structural habitat factors; PC1 loadings) at the trapping plots (Table 3, Figure 6c), a habitat property of especially the fire area (cf. Figure 3). In contrast, in autumn, probability of infection was higher in unburned reference forests (Table 2) and increased with increased habitat complexity (Table 3, Figure 6c). Among the vegetation and structural habitat factors (cf. high PC scores in Figure 3), it was mainly high vegetation cover and multi‐layered shrub and tree canopies that decreased PUUV infection probability in spring. In contrast, absence/low cover of nongrass related vegetation variables and high cover of boulders and high number of snags (cf. low PC scores in Figure 3) increased PUUV infection probability in spring (Figure 6).

**Table 2:** Best generalized linear mixed model on the effect of bank vole density, vole weight, season, and forest type as well as their interaction (×) on PUUV infection probability of bank voles

Estimate	Estimate	Standard error	*z*‐value	*p*‐value
Fixed effects				
Intercept	−3.08	0.64	−4.82	<.001
Vole weight	0.13	0.02	6.90	<.001
Bank vole density	0.18	0.09	2.05	<.05
Autumn	−0.69	0.51	−1.34	
Clear‐cut	0.54	0.67	0.81	
Unburnt reference forest	−0.87	0.38	−2.37	<.05
Bank vole density × autumn	−0.21	0.09	−2.37	<.05
Autumn × Clear‐cut	−0.00	0.73	−0.01	
Autumn × Unburnt reference forest	1.42	0.45	3.16	<.01
	**Variance**	**Standard deviation**		
Random effects				
Trapping plot	0.00	0.00		
Year	0.00	0.00		

**Table 3:** Best generalized linear mixed model on the effect of bank vole density, vole age, season, and first principal component (PC1) of the PC analysis as well as their interaction (×) on PUUV infection probability of bank voles

Estimate	Estimate	Standard error	*z*‐value	*p*‐value
Fixed effects				
Intercept	−2.98	0.48	−6.26	<.001
Vole age	0.26	0.02	10.60	<.001
Bank vole density	0.14	0.09	1.61	
Autumn	0.30	0.53	0.56	
PC1	−0.18	0.07	−2.72	<.01
Bank vole density × autumn	−0.15	0.09	−1.66	
Autumn × PC1	0.28	0.08	3.60	<.001
	**Variance**	**Standard deviation**		
Random effects				
Trapping plot	0.02	0.13		
Year	0.00	0.00		

## 4 DISCUSSION

The forest fire in our study area significantly changed vegetation composition and structural habitat factors with cascading effects on small mammal community structure, population dynamics of bank voles, and pathogen prevalence. The species richness of small mammals was low in all three forest types, and we only trapped few other small mammals than bank voles. We did not trap any field voles even though we know that this species was present at least in the nonstudied part of the fire area, as revealed by three specimens speared on branches of pine trees by Great Grey Shrikes (*Lanius excubitor* Linnaeus) in 2011 (personal observation). Field voles are else known to be common on clear‐cuts in boreal Fennoscandia (Hansson, 1989, 2002). Despite the abundance of weevils in the fire area (Johansson et al., 2011), we did not trap any shrews there either. In fact, after the first postfire year, the fire area was a system comprised by a single small mammal species, viz. the bank vole.

The autumn density of bank voles was higher in clear‐cuts than in unburnt old‐growth forests, a result that is consistent with previous findings in northern Sweden (Ecke et al., 2002; Hansson, 1979). Bank voles in mature forests in northern Sweden largely feed on forbs, fungi, berries, and tree lichens (Hansson, 1979). As evident from our sampling of vegetation, forbs, berries, and tree lichens were largely destroyed by the severe fire, which likely explains why bank vole density was lower in the fire area compared to the unburnt forests. In North America, the generalist deer mice are more common in clear‐cuts and clear‐cut‐burnt forests than in mature forests, however, with no difference in demography among treatments (Sullivan, Lautenschlager, & Wagner, 1999; Zwolak & Foresman, 2008). In our study, the age distribution among bank voles differed among forest types. As observed previously, clear‐cuts have a high proportion of juveniles, especially in autumn (Ecke et al., 2002; Hansson, 1979); a phenomenon that is likely explained by source‐sink dynamics between clear‐cuts and nearby source forests (Ecke et al., 2002). In our study, the proportion of adult bank voles, especially in the first postfire spring, was high in the fire area. This result implies that these bank voles (a) had survived in the fire area since they were borne before the forest fire occurred or (b) recolonized the fire area after the fire. The latter seems unlikely, since in voles, it is mostly subadults and not overwintered breeders that disperse (Gliwicz, 1989; Myllymäki, 1977).

The weight of overwintered breeders of bank voles did not differ between the fire area and unburnt forests. This indicates that food availability and quality in the fire area were similar. In North America, deer mice in burnt and unburnt forests showed similar weight, with high food availability due to fire‐induced exposure of so far unexploited seed banks as potential explanation (Ahlgren, 1966; Zwolak & Foresman, 2008). Bank voles in the temperate and southern boreal zone largely forage on seeds (Gebczynska, 1983), while this is less common in northern Fennoscandia (Hansson, 1979; Hansson & Larsson, 1978), but also in the latter zone, the rate of change in numbers of bank voles is affected by the availability of spruce and pine seeds (Hörnfeldt, 1994). As bank voles also forage on fungi and insects, (Hansson, 1979), the plentiful weevils found in our fire area (Johansson et al., 2011) together with the high number of observed shedded pine seeds and ground‐living fungi (personal observation) might not only have been a highly available but also high quality food for bank voles.

The forest fire was severe and destroyed much of the vegetation in the field, shrub, and tree layer and also the organic soil layer in large parts of the fire area (Johansson et al., 2011). Depending on vegetation type, forest fires generally have a patchy landscape distribution (Hellberg, Niklasson, & Granström, 2004) and show heterogeneity at the local scale, leaving refugia for survival of cryptogams and vascular plants (see Figure 1b and Forsman, 2008). Hence, in our pilot study, bank voles might have survived winter in these refugia and were trapped when foraging in the burnt forest patches. Potential aggregation in the refugia might have increased contact among bank vole specimens and hence increased transmission risk, which could explain the high PUUV prevalence in the fire area. PUUV is horizontally transmitted among bank voles with young bank voles being protected by maternal antibodies (Kallio et al., 2010). The high proportion of bank voles ≥11 months old in the first postfire spring in the fire area and clear‐cuts might therefore explain the high PUUV prevalence in these habitats.

An alternative but not mutually exclusive explanation for the high pathogen prevalence in the fire area might be related to diversity among small mammals (i.e., switch from a multiple rodent species system to a single‐species system); an explanation that would be in line with the amplification effect (the antonym of the dilution effect; Luis, Kuenzi, & Mills, 2018; Ostfeld & Keesing, 2000; Schmidt & Ostfeld, 2001). Species richness was low in all three forest types, but the fire area was the only forest type where only bank voles were trapped after the first postfire year. Reduced species richness of noncompetent hosts (here e.g., gray‐sided vole and wood lemmings) might increase encounter probability among the remaining competent host (here bank vole) individuals. The high PUUV prevalence in spring in the fire area and clear‐cuts might hence be induced by high contact rate among susceptible bank voles.

We also argue that environmental complexity might have decreased infection probability in voles in our study, as revealed by the negative relationship between PC1 loadings and infection probability irrespective of forest type. High PC1 loadings (as observed in unburnt forests) imply high number of micro‐niches for bank voles to avoid direct and/or resource competition with conspecifics. In analogy with the dilution effect, also environmental complexity (incl. community biodiversity and heterogeneity) can reduce encounter rate among competent and susceptible hosts and hence reduce pathogen transmission. However, we also know that habitat complexity and certain habitat properties (especially those providing cover) are a prerequisite for the survival of PUUV infected bank voles (Khalil et al., 2017). PUUV infection probability was positively related to PC1 loadings in autumn. This result might be due to a density effect as bank vole density is an important predictor of PUUV prevalence (Khalil, Ecke, Evander, Magnusson, & Hörnfeldt, 2016; Voutilainen et al., 2012). Bank vole density was significantly higher in autumn than in spring, and in autumn, trapping plots with high complexity are likely to show high bank vole density. In summary, the observed high PUUV prevalence in the fire area might hence not be a forest type effect per se, but rather be explained by the prevailing habitat niches in the area (e.g., cover of large holes and bilberry; cf. Khalil et al., 2017). As we were only able to assess habitat properties once (summer 2010), it would be interesting to repeat this inventory and to study if successional changes, which are likely to be observed in the fire area (Chen, Vasiliauskas, Kayahara, & Ilisson, 2009; Engelmark, 1987; Tiribelli, Kitzberger, & Morales, 2018), have affected both vole density, species richness among small mammals, and PUUV prevalence.

Human PUUV infection risk was probably higher in the unburnt forests compared to the fire area due to higher vole density and higher number of PUUV infected bank voles in spring and autumn in the former forest type. However, given encounter with a bank vole in spring (or encounter with infectious PUUV shedded by bank voles), human risk was higher in the fire area, due to higher PUUV infection probability in forests with low habitat complexity.

Generalist rodents like bank voles and deer mice are hyper‐reservoirs, i.e., they harbor multiple zoonotic pathogens. In this pilot study, we focused on PUUV, but in future studies, research should consider coinfections to evaluate if environmental stress, here forest fire, also increases infection risk of other rodent‐borne pathogens and diseases including those caused and/or facilitated by for example Ljungan virus (Niklasson, Nyholm, Feinstein, Samsioe, & Hörnfeldt, 2006). Since our study included only a single (but major) forest fire area, the generality of our results needs to be validated, especially regarding the effect of forest fire on small mammal community structure and on prevalence of zoonotic pathogens. Here, the major forest fires that struck Sweden in summer 2018 (>60 fires) pose a rare natural experiment to study the hypotheses addressed in our pilot study in a replicated design.

## CONFLICT OF INTEREST

None declared.

## AUTHOR CONTRIBUTIONS

FE and HK conceptualized the study with the help of BH. FE and SANM collected data. SANM and ME analyzed samples. HK, FE, and SANM evaluated the data. FE wrote the manuscript and all coauthors provided comments and approved the manuscript.

## Data Availability

Data on small mammals, structural habitat factors, and PUUV prevalence are available at OSF https://osf.io/6fsy3/.
